# The effect of midwifery led counseling based on Gamble’s approach on childbirth fear and self-efficacy in nulligravida women

**DOI:** 10.1186/s12884-020-03230-1

**Published:** 2020-09-09

**Authors:** Laya Firouzan, Roghieh Kharaghani, Saeedeh Zenoozian, Reza Moloodi, Elham Jafari

**Affiliations:** 1 Department of Midwifery, School of Nursing and Midwifery. Zanjan University of Medical Sciences, Zanjan, Iran; 2 Department of Clinical Psychology, Beheshti Hospital and Zanjan University of Medical Sciences, Zanjan, Iran; 3 Substance Abuse and Dependence Research Center, University of Social Welfare and Rehabilitation Sciences, Tehran, Iran

**Keywords:** Childbirth fear, Childbirth self-efficacy, BELIEF protocol, Iran

## Abstract

**Background:**

Studies show that childbirth fear is a common problem among Iranian women. Therefore, most Iranian women prefer caesarean section for giving birth. This study investigated the effectiveness of a psychoeducational intervention by midwives (birth emotions - looking to improve expectant fear (BELIEF)) on decreasing childbirth fear and self-efficacy among first-time pregnant women who were afraid of giving birth.

**Methods:**

A number of 80 pregnant women participated in the study. They had received a score of ≥66 on the Wijma delivery expectancy/experience questionnaire. They were randomly assigned into two groups: intervention (*n* = 40) and control groups (n = 40). The intervention group received two face-to-face counseling sessions based on the BELEF protocol in the 24th and 34th weeks of pregnancy. Between these two sessions, it also received eight telephone-counseling sessions once a week. The control group only received the prenatal routine care. The outcome measures were childbirth fear, childbirth self-efficacy, and childbirth preference.

**Results:**

The intervention group showed significantly more reduction in childbirth fear and more increase in childbirth self-efficacy compared to the control group. In addition, more women in the intervention group reported that they preferred to give normal vaginal birth than women in the control group.

**Conclusion:**

The BELIEF protocol could be an effective approach in reducing childbirth fear and increasing childbirth self-efficacy among first-time pregnant women who are afraid of giving birth.

**Trial registration number:**

IRCT20101219005417N3, Date of Registration: 19-12-2018.

## Background

Increasing the number of normal vaginal births and decreasing caesarean sections is an important aim in all healthcare systems, including Iran [1]. However, a recent meta-analysis study in Iran reported that 48% of Iranian women choose caesarean section [2]. Studies show that fear of giving birth is the most common reason for caesarean section among Iranian women [1, 2]. This is even more severe in first-time pregnant women. For example, Matinnia et al., [3] reported that 62.6% of first-time pregnant women prefer caesarean section for giving birth and among them 48.2% experience sever childbirth fear. These findings are in line with studies in other countries that indicate childbirth fear is an important factor for choosing caesarean section for giving birth [4–6].

It seems that childbirth fear has increased in recent years [7]. Its prevalence is around 30% in Italian and Swedish women [8]. Studies shows that childbirth fear is prevalent among Iranian women too. For instance, Mortazavi et al. [9] found out that 20% of Iranian women have a moderate fear and 6% a sever fear of giving birth. Andaroon et al. [10] reported that 50.90% of pregnant women experience childbirth fear.

Some studies have shown that there is an association between childbirth fear and caesarean section [11, 12]. Childbirth fear reduces a mother’s self-efficacy for pregnancy and childbirth. Thus, Iran’s Ministry of Health implemented a plan to increase the percentage of normal vaginal birth [13]. However, a recent evaluation of the plan indicated that although caesarean section has decreased in the public hospitals, it has simultaneously increased in the private hospitals [14]. In other words, women who prefer caesarean section, now refer to private hospitals since the beginning of the national plan. Thus, childbirth fear is still prevalent same as before. So, it seems that such a national healthcare planning should consider psychological interventions that reduce childbirth fear and increase childbirth self-efficacy in mothers [15].

There are some approaches to assist women with childbirth fear. For example, in Sweden, obstetrics’ departments have expert teams to help women with severe childbirth fear. Their intervention includes two to four counselling sessions with the spouse, relaxation trainings, a visit to the labor ward and an individualized birth plan [16]. After these counselling sessions, many afraid pregnant women who preferred to undergo caesarean section did not desire to do so as much as before [17, 18].

In 2013, a group of Australian researchers developed a midwife led psychoeducational approach called birth emotions - looking to improve expectant fear (BELIEF) to target childbirth fear [19]. BELIEF is a telephone-counseling psychoeducational approach that is offered by midwives. It emphasizes on expectations and emotions about childbirth fear, expression of feelings, and it helps women to identify and work through the distressing components of childbirth. Some studies show that BELIEF can reduce childbirth fear. Toohill et al. [20] stated that the afraid pregnant women have less childbirth fear and depressive symptoms after this intervention. Another study on women with high childbirth fear indicated that the overall caesarean section rates decrease after undergoing BELIEF clinically [21]. In addition, BELIEF seems to be a cost-effective approach [22].

To our knowledge, there is no study regarding the effectiveness of psychoeducational interventions on childbirth fear among Iranian women. Hence, this study investigated the effectiveness of BELIEF intervention on first-time pregnant Iranian women with high childbirth fear.

## Method

We categorized all the antenatal clinics of Zanjan city, Iran, into three regions based on socio-economic variables. Then, two clinics were randomly selected from each region. From February to September 2019, 171 first-time pregnant women referred to these public antenatal clinics.

First-time pregnant women who were between 18 to 35 years old, could speak and read Persian, had a single fetus, and scored 66 or above on the Wijma delivery expectancy/experience questionnaire (W-DEQ) [23] were selected to participate in the study. Those who had any history of infertility, and mental or physical chronic diseases were excluded.

### Sample size

The sample size was estimated as 34 participants for each group according to the mean and standard deviation of childbirth fear scores for the intervention (36.3 ± 8) and control groups (30.6 ± 8.6) of a previous research on Iranian first-time pregnant women who were afraid of giving birth [24], power = .80, and error of type 1 = .05. Predicting 20% attrition rate, the sample size of 40 was calculated for each group.

### Data collection

We initially recruited 171 first-time pregnant women who were in 20th to 23rd weeks of pregnancy. They were informed about the research by the midwives and those who signed a written consent were recruited. At first, they answered the W-DEQ [23]. 91 women were excluded (32 women did not show childbirth fear, and 59 women did not meet the inclusion criteria). Thus, 80 first-time pregnant women with childbirth fear participated in the study.

The participants were randomly assigned into intervention (*n* = 40) or control groups (n = 40). We did the randomization with four-way blocks. The randomization code was produced by a web-based randomization software. The assessors and data analyzer were not aware of the group assignment. 12 women (five in the intervention group and seven in the control group) dropped out of the study because of immigration, preterm childbirth, fetus death, and occurrence of diabetes (Fig. . 1). All women answered the demographic information questionnaire, W-DEQ [23], and childbirth self-efficacy inventory [25] at pretest and post-test.

**Fig. 1:**
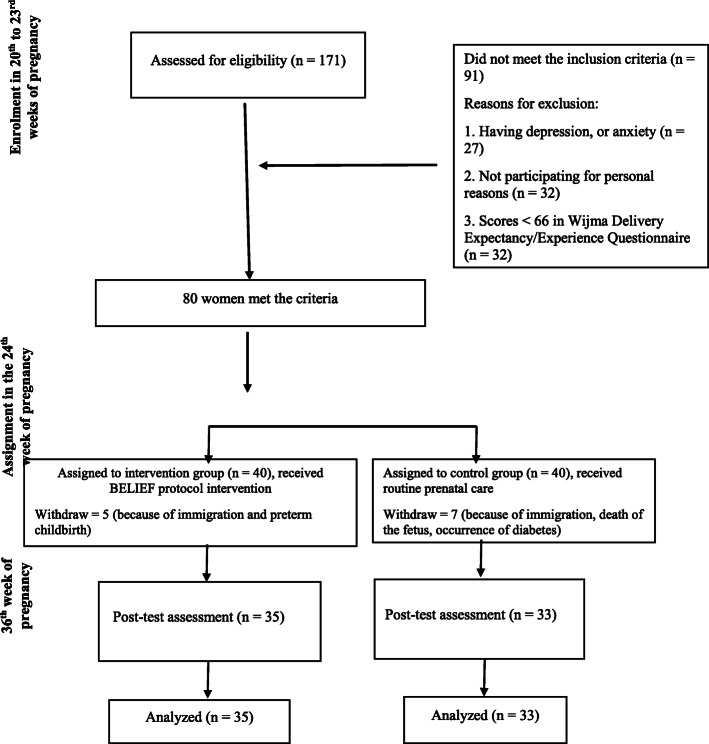
Flow diagram of the study

2.2.1. The sociodemographic questionnaire included age, educational status, and occupation.

2.2.2. Childbirth preference was assessed with the following question: “Which method do you prefer for giving birth? A) normal vaginal birth, B) caesarean section”.

2.2.3. WDEQ-A: This questionnaire assesses the intensity of emotions related to childbirth expectations. It consists of 33 items on a six-point Likert scale (0 = do not agree; 5 = totally agree) [23]. The total score ranges from 0 to 165. Higher scores reflect greater level of childbirth fear. A score ≥ 66 reflects severe childbirth fear. Women are asked to answer the questions while imagining how labor and delivery are going to be, and how they expect it to feel. Items 2, 3, 6, 7, 8, 11, 12, 15, 19, 20, 24, 25, 27, and 31 are reverse-scored. Reliability and validity of WDEQ-A have been demonstrated in different populations [23, 26], as well as Iranians [27]. In our study, internal consistency of the WDEQ-A was .86.

2.2.4. Childbirth self-efficacy inventory: This 62-item questionnaire assesses maternal confidence in coping abilities during labor [25]. Women are asked to answer the questions based on a 10-point Likert scale. It has four subscales: (1) Items 1–15 measure outcome expectancy active labor (Outcome-AL); (2) Items 16–30 assess self-efficacy expectancy active labor (Efficacy-AL); (3) Items 31–46 measure expectancy second stage (Outcome-SS); (4) items 47–62 measure self-efficacy expectancy second stage (Efficacy-SS). The two total scores are: (i) the total childbirth outcome expectancy score (outcome total), which is calculated by summing the Outcome AL and Outcome SS scale scores and (ii) the total self-efficacy expectancy score (efficacy total), which is calculated by summing the Efficacy AL and Efficacy SS scale scores. Higher scores reflect greater level of childbirth self-efficacy. Validity and reliability of the Persian version [28] of the Childbirth self-efficacy inventory has been established. In our study, the internal consistency of the scale was .98.

### Procedure

The first author gave two face-to-face counseling sessions in the 24th and 34th weeks of pregnancy to the participants in intervention group (she is a midwife). Between these two sessions, the intervention group received eight telephone-counseling sessions once a week. We used the BELIEF approach which is a telephone counseling psychoeducational approach offered by midwives [19]. It helps women to develop individualized support for the present and near future, affirming that negative events can be coped with simple problem-solving skills. The third and fourth authors trained the first author on how to conduct the BELIEF approach. The fourth author randomly listened to the record of the sessions to assure that the intervention is in accordance with the BELIEF protocol (Fig. 1).

### Data analysis

We analyzed the data using the statistical package for social sciences (SPSS) software version 24. We used descriptive statistics to describe the demographic characteristics of the participants. The two groups were compared regarding the socio-demographic characteristics using independent t-test and Chi-square test. Shapiro-Wilk test indicated that the variables have a normal distribution (*p* value ranged from 0.12 to 0.34). Preliminary checks were conducted to ensure that there was no violation of the assumptions of normality, linearity, homogeneity of variances, homogeneity of regression slopes, and reliable measurement of the covariate. Thus, one-way between-groups analysis of covariance was used to determine the differences between the two groups on the childbirth fear, childbirth self-efficacy, childbirth preference.

### Ethical considerations

This study was registered in the registry for clinical trials (IRCT20101219005417N3). The ethics committee of our university approved the research procedure (IR.ZUMS.REC.1397.025). All participants provided a signed written informed consent and they could leave at any stage of the research.

## Results

The intervention group’s mean of age was 26.27 ± 4.48 years old. The control group’s mean of age was 25.87 ± 4.58 years old. The means of their husbands’ ages were 30.87 ± 4.46 and 29.15 ± 3.69 years old for the intervention and control groups, respectively. There was no difference between the two groups regarding their own age (*t* (66) = 1.38, *p* = 0.17), and their husbands’ age (t (66) = 1.42, *p* = 0.08).

The preliminary analysis indicated that there were no differences between the two groups in terms of their own educational status (*x*^2^ (2, *N* = 68) = 0.058, *p* = 0.80), their husbands’ educational status (*x*^2^ (2, *N* = 68) = 0.23, *p* = 0.62), employment status (*x*^2^ (2, N = 68) = 0.098, *p* = 0.95), and economic status ((*x*^2^ (2, N = 68) = 0.80, *p* = 0.26). In addition, they were not different regarding pre-test scores of childbirth self-efficacy inventory (*t* (66) = 1.37, *p* = 0.17), and childbirth preference (*x*^2^ (2, N = 68) = 0.000, *p* = .99) (Table 1). However, the intervention group got higher scores on W-DEQ-A (*t* (66) = 2.33, *p* = 0.02) than the control group at pre-test assessment (Table 1). Twelve (15%) participants left the study before providing post-test data. They were not different from those who provided complete data regarding baseline variables (all *p*-values > .24–.81), implying that attrition did not bias the results.

**Table 1:** Demographic characteristics and childbirth preference at pre-test and post-test assessment of intervention (n = 35) and control groups (n = 33)

	Intervention group	Control group	*p*
	N (%)	N (%)	
***Educational status***			
Diploma	24 (68.5%)	23 (69.7%)	.8
Bachelor or higher	11 (31.5%)	10 (30.3%)	
***Husbands’ educational status***			
Diploma	24 (68.5%)	25 (75.75%)	.62
Bachelor or higher	11 (31.5%)	11(24.25%)	
***Employment status***			
Housewife	31 (88.57%)	29 (87.87%)	.95
employee	4 (11.43%)	4 (12.12%)	
***Economic status***			
Low income	7 (20%)	10 (30.30%)	.26
Moderate income	22 (62.85%)	17 (51.51%)	
High income	6 (17.15%)	6 (18.18%)	
***Childbirth preference at pre-test***			
Normal vaginal birth	22 (62.85%)	21 (63.63%)	.99
Ceasarion Section	13 (37.15%)	12 (36.36%)	
***Childbirth preference at post-test***			
Normal vaginal birth	29 (82.85%)	19 (57.57%)	.02
Ceasarion Section	3 (8.57%)	12 (36.36%)	
Have not decided yet	3 (8.57%)	2 (6.06%)	

### Intervention effects on childbirth fear

To investigate the effect of BELIEF protocol on childbirth fear, a one-way between-groups analysis of covariance was conducted to test whether the intervention group showed a significant decrease in childbirth fear (measured by W-DEQ-A) compared to the control group (Table 2). After adjusting for the pretest scores, there was a significant difference between the intervention and control groups on post-test scores of W-DEQ-A (F (1, 65) = 100.42, *p* = .0001, partial eta squared = .60). In other words, the intervention group got lower scores on W-DEQ-A at post-test than control group (Table 2), indicating that the BELIEF protocol was effective in decreasing childbirth fear.

**Table 2:** Comparing the two groups on Wijma Delivery Expectancy/Experience Questionnaire-A and Childbirth Self-Efficacy Inventory scores

	Pre-test ***M (SD)***	Post-test ***M (SD)***	***p***	Effect size
***Wijma Delivery expectancy/Experience Questionnaire-A***				
Intervention group	79.8 (12.73)	48.57(16.88)	.0001	.60
Control group	73.48 (9.1)	77.03 (10.72)		
***Childbirth Self-Efficacy Inventory***			.0001	.46
Intervention group	347.74 (98.57)	470 (88.65)		
Control group	384.150 (121.33)	327.21(125.37)		

### Intervention effects on childbirth self-efficacy

To investigate the effect of BELIEF protocol on childbirth self-efficacy, a one-way between-groups analysis of covariance was conducted to test whether the intervention group showed a significant increase in childbirth self-efficacy (measured by childbirth self-efficacy inventory) compared with the control group (Table 2). After adjusting for the pre-test scores, there was significant difference between the two groups on post-test scores of childbirth self-efficacy inventory (F (1, 65) = 57.23, *p* = .0001, partial eta squared = .46). In other words, the intervention group got higher scores on this inventory than the control group (Table 2), suggesting that BELIEF intervention effectively improved childbirth self-efficacy of the afraid pregnant women.

### Intervention effects on childbirth preference

After the intervention, more women in the intervention group (*n* = 29 (82.85%)) stated that they preferred to give normal vaginal birth than women in the control group (*n* = 19 (57.57%)), (*x*^2^ (2, *N* = 68) = 7.63, *p* = 0.02). Thus, the BELIEF intervention was effective in increasing the desire of pregnant women to do normal vaginal birth (Table 2).

## Discussion

Childbirth fear is a prevalent problem among pregnant women. In our study, 80 of 171 first-time pregnant women who had referred to the studied public antenatal clinics (46.78%) experienced severe childbirth fear, and one third of the afraid women preferred caesarean section at pretest. This reflects the necessity of implementing psychoeducational interventions to reduce childbirth fear among the afraid pregnant women.

Our results showed that a brief telephone-counseling psychoeducational intervention (BELIEF protocol) provided by midwives during 24th to 34th weeks of pregnancy is significantly effective in reducing women’s childbirth fear and improving childbirth self-confidence. In addition, they showed that after BELIEF intervention more women prefer normal vaginal birth. However, women in the control group had a greater level of childbirth fear and less childbirth self-efficacy at the post-test compared to the pre-test. These results imply that without a psychoeducational intervention, childbirth fear would even intensify in the weeks leading up to pregnancy.

Our findings are in line with the previous studies that show that BELIEF intervention effectively decreases childbirth fear, depression symptoms, and caesarean section rate, and improve women’s self-confidence about labor [20, 21]. In addition, they are consistent with researches that have reported that other psychological interventions are fruitful in reducing childbirth birth fear among the afraid pregnant women [16–18].

It seems that the BELIEF protocol improves women’s attitudes about their ability to cope with normal physiological and emotional difficulties of labor and thereby reduces childbirth fear. Also, this intervention helps women to understand and accept the unpredictable and painful nature of childbirth. To our knowledge, this is the first study in Iran and third worldwide that explores the effectiveness of BELIEF protocol on childbirth fear. The previous two studies had been done in Australia [20, 21]. Thus, further research is needed to investigate the effectiveness of the BELIEF intervention on childbirth fear in different populations.

A positive aspect of our research was that we assessed the effectiveness of BELIEF protocol on pregnant women who were afraid of giving birth. This protocol focuses on the counseling role of midwives in the prenatal care. Since providing specialized psychological and psychiatric services is not possible for all pregnant women, providing such psychoeducational approaches by midwives would be a logical and cost-effective strategy. In the BELIEF protocol, the midwife helps a woman to explore the origins of her childbirth fear, and neutralize impacts of negative events of previous childbirth experiences. In addition, the midwife informs a pregnant woman of her birth options and helps her to develop strategies for a positive birth experience.

### Limitations

These results should be interpreted having the limitations in mind. First, we only used self-report questionnaires to assess the outcome variables. Using face-to-face deep interviews helps researchers to measure childbirth fear and self-confidence more precisely. Second, we only assessed childbirth preference at post-test and we were not aware of the impact of the intervention on reducing caesarean section rate. Thus, future research should also explore the impact of the BELIEF intervention on caesarean section rate.

## Conclusion

Our study shows that a psychoeducational counseling intervention by midwives could be effective in reducing childbirth fear. This shows that it is important to include brief psychoeducational programs in the trainings of midwifes. In addition, screening of the afraid pregnant women is recommended to identify those who suffer childbirth fear and prefer caesarean section because of it. Finally, further researches is needed to explore the effectiveness of BELIEF on the reduction of caesarean section rate among Iranian women.

## Data Availability

Zanjan University of Medical Sciences has approved and supported that only researchers of the manuscript will have access to the dataset, so the data used in this study is not available for public view. Still, requests can be written officially to the university.

## Abbreviations

BELIEF: birth emotions - looking to improve expectant fear
SPSS: Statistical Package for the Social Sciences
WDEQ: Wijma Delivery Expectancy/Experience Questionnaire
